# A preliminary analysis of genome structure and composition in *Gossypium hirsutum*

**DOI:** 10.1186/1471-2164-9-314

**Published:** 2008-07-01

**Authors:** Wangzhen Guo, Caiping Cai, Changbiao Wang, Liang Zhao, Lei Wang, Tianzhen Zhang

**Affiliations:** 1 National Key Laboratory of Crop Genetics & Germplasm Enhancement, Cotton Research Institute, Nanjing Agricultural University, Nanjing 210095, PR China

## Abstract

**Background:**

Upland cotton has the highest yield, and accounts for > 95% of world cotton production. Decoding upland cotton genomes will undoubtedly provide the ultimate reference and resource for structural, functional, and evolutionary studies of the species. Here, we employed GeneTrek and BAC tagging information approaches to predict the general composition and structure of the allotetraploid cotton genome.

**Results:**

142 BAC sequences from *Gossypium hirsutum *cv. Maxxa were downloaded  and confirmed. These BAC sequence analysis revealed that the tetraploid cotton genome contains over 70,000 candidate genes with duplicated gene copies in homoeologous A- and D-subgenome regions. Gene distribution is uneven, with gene-rich and gene-free regions of the genome. Twenty-one percent of the 142 BACs lacked genes. BAC gene density ranged from 0 to 33.2 per 100 kb, whereas most gene islands contained only one gene with an average of 1.5 genes per island. Retro-elements were found to be a major component, first an enriched LTR/gypsy and second LTR/copia. Most LTR retrotransposons were truncated and in nested structures. In addition, 166 polymorphic loci amplified with SSRs developed from 70 BAC clones were tagged on our backbone genetic map. Seventy-five percent (125/166) of the polymorphic loci were tagged on the D-subgenome. By comprehensively analyzing the molecular size of amplified products among tetraploid *G. hirsutum *cv. Maxxa, acc. TM-1, and *G. barbadense *cv. Hai7124, and diploid *G. herbaceum *var. *africanum *and *G. raimondii*, 37 BACs, 12 from the A- and 25 from the D-subgenome, were further anchored to their corresponding subgenome chromosomes. After a large amount of genes sequence comparison from different subgenome BACs, the result showed that introns might have no contribution to different subgenome size in *Gossypium*.

**Conclusion:**

This study provides us with the first glimpse of cotton genome complexity and serves as a foundation for tetraploid cotton whole genomesequencing in the future.

## Background

Cotton is the world's most important natural textile fiber and a significant oilseed crop. The cotton genus (*Gossypium *L.) includes approximately 45 diploid species (2*n *= 2*x *= 26) differentiated cytogenetically into eight genome groups (A-G & K), and five allotetraploid species (2*n *= 4*x *= 52) [1]. Diploid *Gossypium *species differentiated approximately 5–10 million years ago (Mya), however, polyploidization is estimated to have occurred more recently 1–2 Mya [2]. All allotetraploids were formed from interspecific hybridization events between an A-genome-like ancestral African species and a D-genome-like North American species. The closest extant relative of the original tetraploid progenitors is the A-genome species *G. herbaceum *L. (A1) and the D-genome species *G. raimondii *(D5) Ulbrich. Of these, four cotton species, including two tetraploids *G. hirsutum *L. (AD)1 and *G. barbadense *L (AD)2, and two diploids *G. herbaceum *L. (A1) and *G. arboreum *L. (A2) were independently domesticated for fiber.

Upland cotton has the highest yield, and based on the importance of fiber, over 95% of the annual worldwide cotton crop is derived from *G. hirsutum *L., upland cotton, and the extra-long staple (ELS) or Pima cotton (*G. barbadense *L.) accounts for less than 2% [3]. Two diploid species *G. herbaceum *L. (A1) and *G. arboreum *L. (A2) are planted less often. In cultivated tetraploid cotton species, the D-subgenome plays an important role in genome structure, function and evolution. For example, many quantitative trait loci (QTL) for fiber-related traits have been detected in the D-subgenome of tetraploid cotton [4-9]. D-genome species do not produce spinnable fiber [10]; however important genes or regulators for fiber morphogenesis and fiber properties have been detected in this genome. Based on the above analyses, understanding the contribution of the A- and D-subgenomes to gene expression in the allotetraploids may greatly facilitate fiber trait improvement [11,12]. To attain this goal, decoding cotton genomes will be a foundation to enhance our understanding of the functional and agronomic significance of polyploidy and genome size variation within *Gossypium *[13].

Genome size differences are evident in the tetraploids and their diploid progenitors. The haploid genome size is estimated to be ~980-Mb for *G. raimondii *Ulbrich, ~1.86-Gb for *G. arboreum *L., and ~2.83 Gb for *G. hirsutum *L. [14]. Diploid species variation in DNA content reflects increases and decreases in copy numbers of various repeat families [15], especially retrotransposon-like elements [16]. The method most appropriate for elucidating whole-genome sequence information in cotton is either BAC-by-BAC sequencing or gene-enrichment approaches. A pilot study by the U.S. Department of Energy Joint Genome Institutes [17] has been initiated to generate the whole-genome shotgun sequence of *G. raimondii*. Meanwhile, gene-enrichment techniques such as methylation filtration and C_o_t-based cloning have also been used to compare *G. raimondii, G. arboreum, G. hirsutum*, and *G. barbadense *(B. Scheffler, Workshop communication).

The whole-genome sequence analysis of *G. hirsutum *will undoubtedly provide the ultimate reference and resource for structural, functional, and evolutionary studies of the species that accounts for > 95% of world cotton production. Prior to large-scale sequencing of tetraploid *G. hirsutum *genomes, a microcolinearity analysis of a few pairs of homoeologous BACs was completed, and indicated that sequence conservation of homoeologous BACs was high in both intergenic and genic regions [14]. In addition, Grover et al. (2007)[18] suggested size differences between homoeologous BACs was attributed to differential accumulation of retroelements.

The GeneTrek approach has been proposed as an efficient way to evaluate the general properties of any genome [19,20] and has been successfully applied to predictions regarding components of the maize genome [21]. To better understand the general composition and structure of the tetraploid cotton genome, in the present paper, we also employed GeneTrek and BAC tagging information approaches to analyze. This methodology facilitated our evaluation of the structure and composition of the allotetraploid genome based on 142 *G. hirsutum *cv. Maxxa BAC clones downloaded from the National Center for Biotechnology Information (NCBI) [22]. The study provided us the first glimpse at cotton genome complexity, and the results indicated that the gene distribution in cotton genome is uneven with gene-rich and gene-free regions, and rich in repetitive elements. Introns might have no contribution to different subgenome size in *Gossypium*, and a two-fold genome difference between A- and D-subgenomes, which might largely be attributed to large amplifications of transposable elements in low-density gene or gene-free regions.

## Results

### Confirmation of 142 BACs origin

Due to the fact that 142 BACs were result from a mistake first submitted as part of the maize sequencing project by the Genome Sequencing Center, Washington University School of Medicine and further corrected as *G. hirsutum *cv. Maxxa BAC clones, we downloaded these BACs from the National Center for Biotechnology Information (NCBI) [22] and confirmed their origin by developing BAC-SSR markers from 142 BAC sequences.

Each BAC was scanned for dinucleiotide to hexanucleiotide repeats of at least 18 bp in length. A total of 694 microsatellite sequences were detected. Among them, 208 SSRs were dinucleotides, 118 trinucleotides, 69 tetranucleotides, 80 pentanucleotides and 219 hexanucleotides. In addition, 578 SSR primer pairs were developed and used to detect the amplification ability in *G. hirsutum *cv. Maxxa, and our two mapping parents, *G. hirsutum *acc. TM-1 and *G. barbadense *cv. Hai7124. Among them, all 578 primer pairs amplified expected fragment sizes in *G. hirsutum *cv. Maxxa, and 161 primer pairs from 79 BACs amplified polymorphisms between TM-1 and Hai7124, yielding a 27.85% polymorphic rate. Both the high-level transferability among *G. hirsutum *cv. Maxxa, acc. TM-1, and *G. barbadense *cv. Hai7124 and the high-level polymorphism between TM-1 and Hai7124 indicated that these 142 BAC sequences must be from Maxxa genome. Further, these genomic SSR markers also have potential for use in future cotton genomics and molecular breeding. The newly developed SSR primer sequences, Genbank accession numbers, repeat motifs and numbers, expected product size, and polymorphic data between TM-1 and Hai7124 are presented in additional file 1.

### Global analysis of genome structure and composition of tetraploid cotton

#### Gene annotations

Using the sequence information of the 142 BACs spanning 14.2 Mb of the cotton genome, genome structure and composition of tetraploid cotton were analyzed. Comprehensively analyzing the gene prediction results from three *ab initio *gene prediction programs FGENESH, GENEMARK.HMM and GENSCAN+, 3,440 gene models were predicted. Of them, 1,329 (38.6%) were identified repeat components (mostly LTR retrotransposons), which were further analyzed with mobile elements; 1,653 (47.9%) lacked homology to other NCBI protein database species. Furthermore, the putative protein-encoding gene was subjected to BLASTN queries against the cotton EST database released in NCBI and two hundred eight were partially confirmed by EST evidence; and 458 showed homology to other species in the NCBI protein database. Based on significant homology to other species (*e *≤ 10^-10^), 412 gene models were classified as verified gene candidates, with an average gene density of one gene per 34.5 kb. Forty-six gene models were classified as hypothetical proteins. If these gene numbers are extrapolated to the entire tetraploid cotton genome, with an estimated size of 2,500 Mb, tetraploid cotton contains more than 70,000 (verified) genes (Table 1). Details on the annotation of each predicted gene can be found in additional file 2.

**Table 1:** Summary of annotation results for 142 randomly selected cotton BACs

Total number of BACs analyzed	142
Combined BAC lengths	14.2 Mb
Amount of identified repetitive DNA (percentage)	5.7 Mb (40.1%)
Amount of unidentified DNA with predicted ORFs structure (Nos, percentage)	1.4 Mb (1653, 9.9%)
Unidentified ORFs that show collinearity with cotton EST database (percentage)	208 (12.6%)
Number of genes with similarity or collinearity support	412
Number of hypothetical genes with low similarity or collinearity support	46
Overall gene density	One gene per 34.5 kb
Number of estimated total cotton genes	More than 70,000

#### Local gene density and distribution

**Figure 1:**
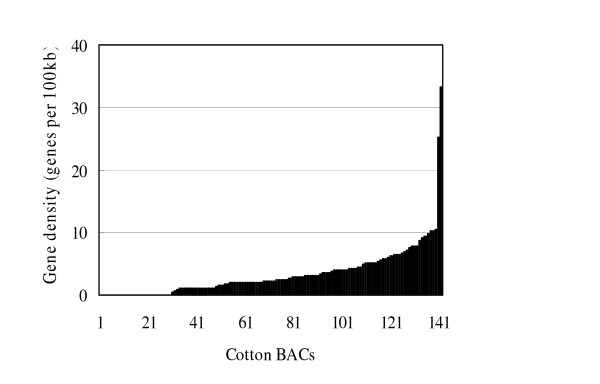
**Gene density variation among BACs.** The BACs are sorted by overall gene density.

Among the 142 analyzed BACs, 30 (21%) did not contain either a verified or hypothetical gene. Furthermore, gene density was estimated as the number of genes on a BAC divided by BAC length. The results showed that on different BACs, gene density varied from 0 to 33.2 per 100 kb (Figure 1, Additional file 3). This indicated uneven cotton gene distribution, and a higher gene density in some regions than others. In AC188398 and AC189045 BACs, 30 and 19 gene models were predicted, respectively, lacking repetitive elements.

#### Gene islands

**Figure 2:**
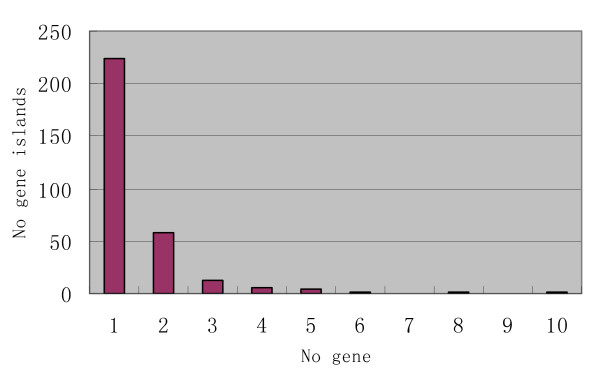
Gene distribution in gene islands.

The number of genes per one gene island can be determined by gene distribution in gene-rich regions. The number of genes on one gene island is counted according to the following criterion: the identifiable repetitive sequences in the intergenic region between two neighboring genes must be less than 5 kb [21]. Furthermore, genes at either end of a BAC or gap within one BAC are discarded from the analysis because one boundary of the gene island is not defined. Based on this criterion, 309 gene islands from one to ten genes (both verified and hypothetical genes) were resolved (Figure 2). Two hundred twenty-four of 309 (72.5%) gene islands contained only one gene with an average of 1.5 genes per gene island.

#### Tandem duplication of genes

Thirty gene islands contained more than two genes, and in those islands, several types of tandem duplication genes encoding the same function were identified (see Additional file 4). According to the molecular function classification of these duplication genes, most were related to binding, such as sar1 GTP-binding secretory factor, ire kinase, RNA-binding protein 10, swi2 snf2-like protein, succinate dehydrogenase flavoprotein alpha subunit, adenylate kinase, and sll2 protein. Other genes functioned in catalytic activities, including genes coding ornithine carbamoyltransferase, glucose-methanol-cholineoxidoreductase family protein, adenylosuccinate lyase, protein phosphatase-5, protein kinase family protein, methylmalonate-semialdehyde dehydrogenase, calcineurin-like phosphoesterase family proteins and serine carboxypeptidase ii. Additional genes were determined to serve in transporter activities such as plasma membrane intrinsic proteins, structural molecule activity such as 50s ribosomal protein l15, and unknown molecular function, such as growth-regulating factor 1, among others. Several disease-resistant gene clusters resided in AC187066, AC190836 and AC202830 BACs. These specific gene clusters presumably accumulated more mutations in both coding and upstream promoter regions to favor a broader response to pathogen attack [23]. Several QTLs related to *Verticillium*-resistance [24] were also found in these regions, but warrants further investigation.

#### Mobile elements analysis

RepeatMasker and CENSOR program was first applied to search for repetitive elements. In all, 1,951 mobile elements with a total length of 1,468,873 bp were predicted (Table 2). Retro-elements were a major component and accounted for 93.9% of the predicted elements with LTR/gypsy comprising 61.1%, LTR/copia 31.2% and LINE elements 1.5%. Four types of DNA transposons were identified, including hobo-Activator (0.3%), En-Spm (3.6%), MUDR-IS905 (0.6%) and Tourist/Harbinger (0.2%). DNA transposons accounted for 4.7% of all predicted elements. A scan of the 142 cotton BACs predicted 0.2% RC/Helitron and 1.1% unclassified mobile elements. Three hundred forty-four intact LTR retrotransposons were predicted and identified by LTR_finder software, leading to an additional 656,779 bp of LTR repetitive sequences. Including the 3,526,152 bp sequence length repeats identified via gene models, mobile DNA accounted for at least 5.7 Mb or 40% of the BAC sequences (Table 1).

**Table 2:** Types of Transposable elements in cotton genome

Retroelements		No. elements	Length occupied (bp)	Percentage
	Lines(L1/CIN4)	30	7242	1.50%
	LTR elements(Ty1/Copia)	608	384327	31.20%
	LTR elements(Gypsy/DIRS1)	1192	1052315	61.10%
	LTR elements(unclasiffied)	2	396	0.10%
	Total	1832	1444280	93.90%
DNA transposons				
	hobo-Activator	6	1525	0.30%
	En-Spm	70	16371	3.60%
	MUDR-IS905	12	961	0.60%
	Tourist/Harbinger	5	1504	0.20%
	Total	93	20361	4.70%
RC/Helitron		4	238	0.20%
Unclassified		22	3455	1.10%
Total		1951		100%

#### Sequence analysis of gene-free BACs

Among the 142 analyzed BACs, 30 showed the complete absence of genes. To further investigate the content of such genomic regions, seven of the 30 BACs with complete sequence assembly were selected. These regions were largely comprised of LTR retrotransposons and were all the primary components of all seven BACs. In retrotransposon types, a number of fragmented *gypsy*-like elements were found in a large "*gypsy*-landing pad", indicating *gypsy*-like retroelements were substantial components of gene-free BACs. All retroelements identified in AC187849, AC194319, AC189931 and AC190814 were *gypsy *elements. The second component of retroelements was a *copia*-like element. The seven gene-free BACs showed the absence of any LINE element (Table 3). Most LTR retrotransposons in the seven BACs were truncated, and only 18 had two intact LTRs and target site duplications (TSD). Most of these LTRs were organized in nested structures.

**Table 3:** Repetitive elements in 7 gene-free BACs

BACs	Length (bp)	No. mobile elements	Gypsy-like	Copia-like	RC/helitron	No. intact LTRs
AC187206	87776	22	16	6		2
AC187396	95093	16	8	7	1	0
AC187849	103016	55	55			5
AC189743	110888	17	11	6		2
AC189931	103756	28	28			2
AC190814	103822	18	18			1
AC194319	77717	40	40			6

### Comparative analysis of genome structure and composition between A- and D- subgenome chromosomes

#### Temporal mapping of 70 BACs based on SSRs

**Figure 3:**
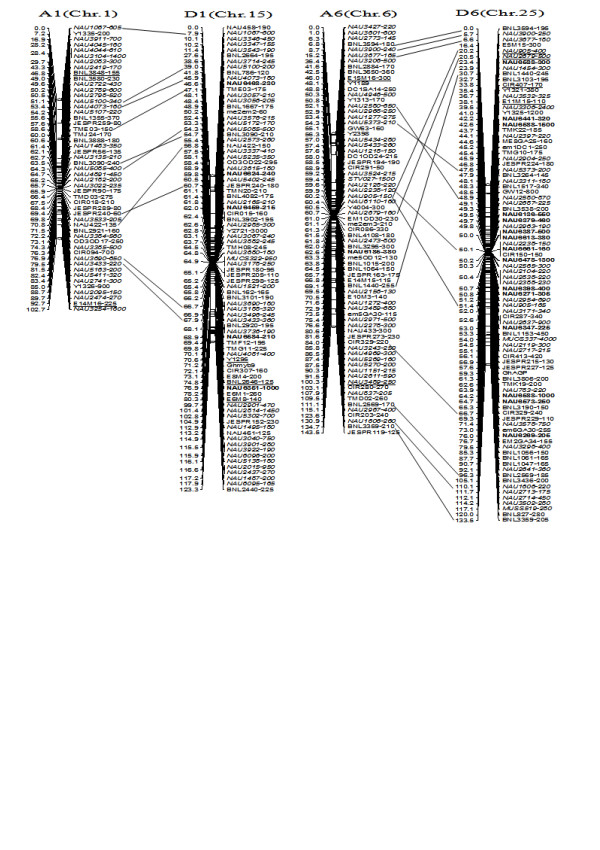
**A updated genetic map of A1/D1 and A6/D6 homoeologous pairs**. Note: Genetic map was constructed using a BC_1 _population obtained from the interspecific cross: *G. hirsutum *L. acc. TM-1 × *G. barbadense *L. cv. Hai7124. Chromosomes and linkage groups are arranged by homoeologous pairs and their corresponding conventional chromosome numbers denoted in bracket. Positions of loci are given in centiMorgans. Fragment sizes from Hai7124 allele (in base pair) are given next to the marker name, and the marker loci unmarked fragment size indicate tagged genes unpublished. Deviated loci are underlined. All SSR-derived BAC clones markers are indicated in bold. Homoeologous loci identified are connected by a bar.

**Figure 4:**
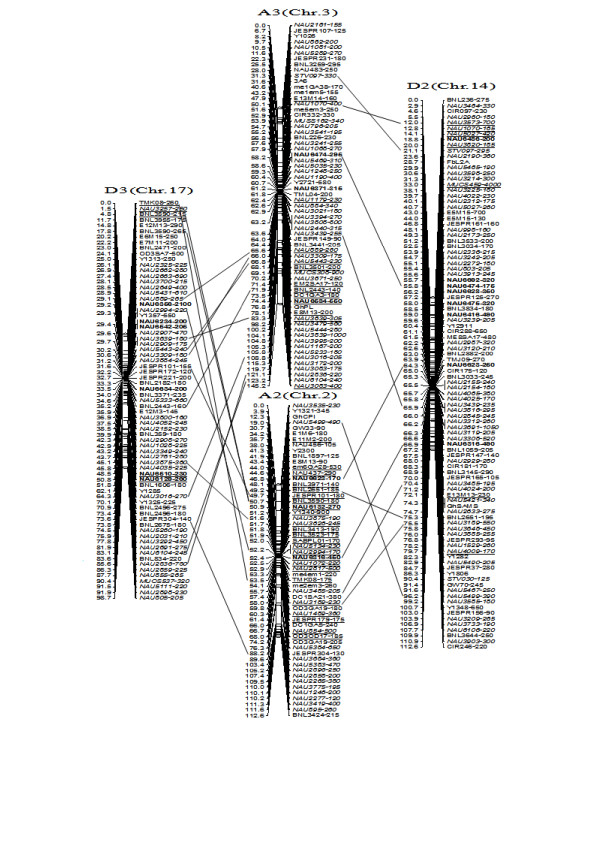
**A updated genetic map of A2/D2 and A3/D3 homoeologous pairs**. All legends are same as described for Figure 3.

**Figure 5:**
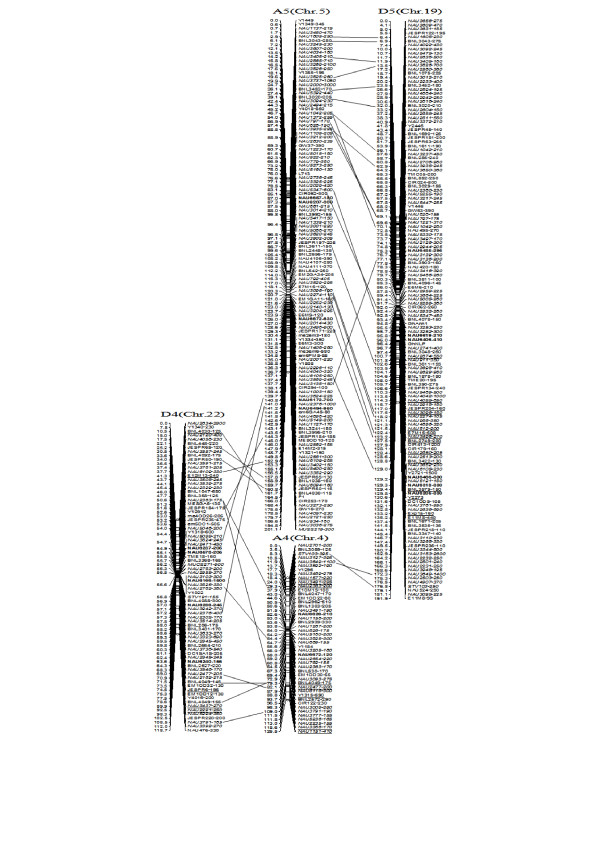
**A updated genetic map of A4/D4 and A5/D5 homoeologous pairs**. All legends are same as described for Figure 3.

**Figure 6:**
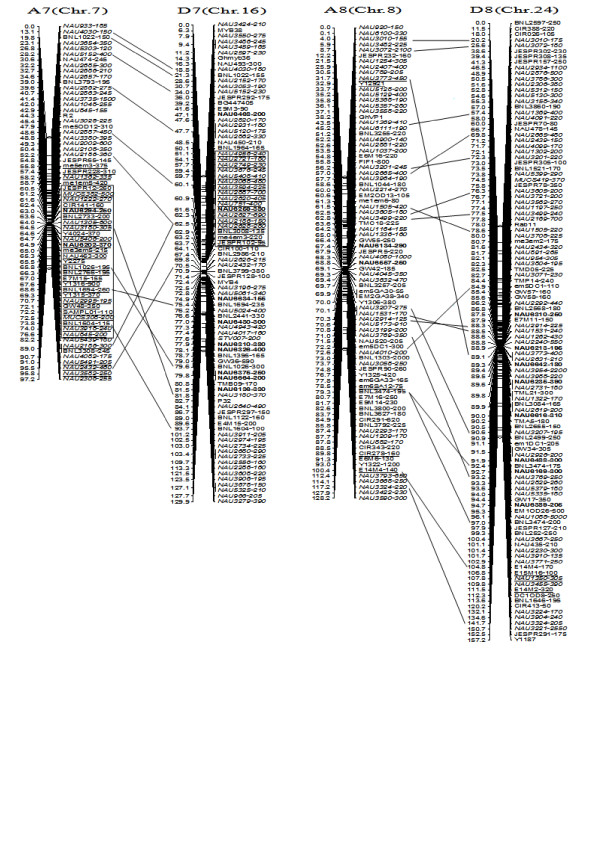
**A updated genetic map of A7/D7 and A8/D8 homoeologous pairs**. All legends are same as described for Figure 3. Deviated interval in A7 and D7 is boxed.

**Figure 7:**
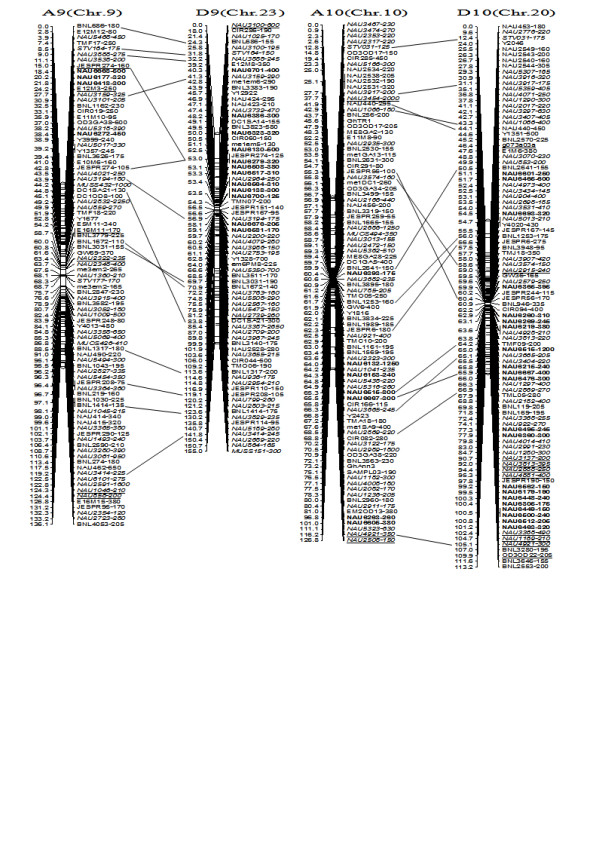
**A updated genetic map of A9/D9 and A10/D10 homoeologous pairs**. All legends are same as described for Figure 3.

**Figure 8:**
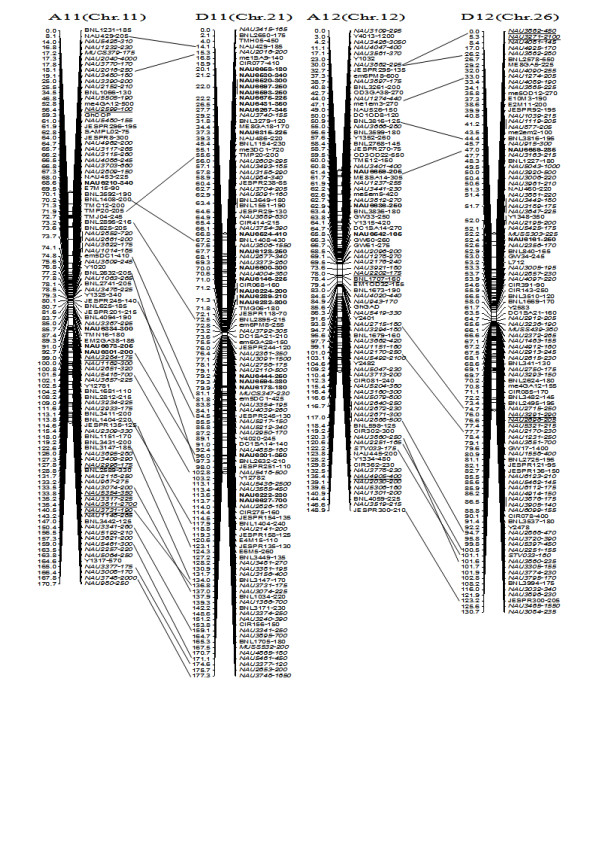
**A updated genetic map of A11/D11 and A12/D12 homoeologous pairs**. All legends are same as described for Figure 3.

**Figure 9:**
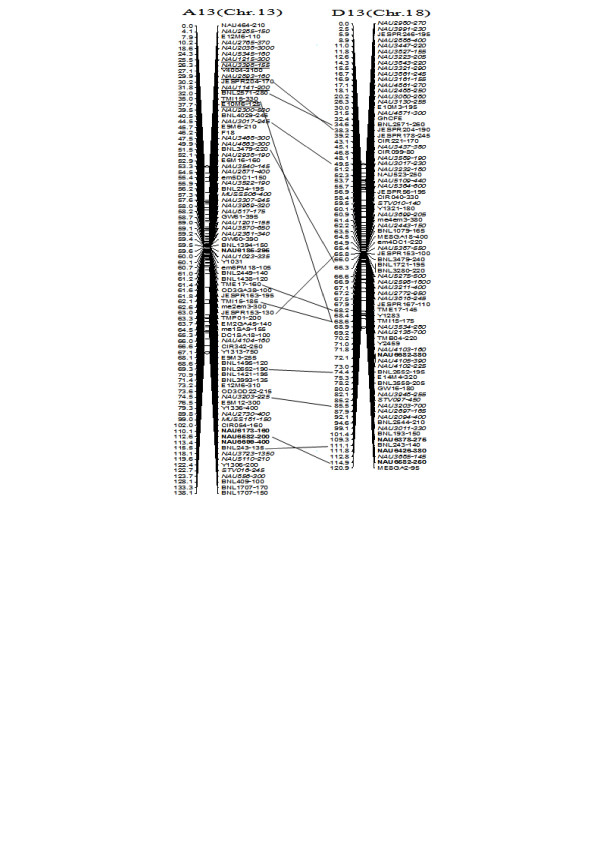
**A updated genetic map of A13/D13 homoeologous pairs**. All legends are same as described for Figure 3.

To compare the A- and D-subgenome chromosome structures and compositions, the BACs must be anchored into their corresponding subgenome or chromosomes. We firstly based the present research on developed SSR markers and our mapping population. By polymorphism analysis, one hundred sixty-one primer pairs developed from 79 BACs could produce 183 polymorphic loci in the two mapping parents, TM-1 and Hai7124. Because TM-1 was used as the recurrent parent in the backcrossed population, 17 dominant TM-1 loci, (of the 183 polymorphic loci) could not be used to anchor the related-BACs to their corresponding chromosomes. The remaining 166 polymorphic loci, amplified from 144 polymorphic SSR primer pairs were integrated into our previously published map containing 1,790 loci and spanning 3425.8 cM [25]. Subsequently, a new updated genetic map composed of 2,247 loci in 26 linkage groups covering 3540.4 cM with an average inter-marker distance of 1.58 cM was produced (Figure 3, 4, 5, 6, 7, 8, 9). Based on the new integrated genetic map, 166 polymorphic loci developed from 70 BAC clones were anchored to their corresponding chromosomes. Further analysis of 70 BACs with tagged results revealed 18 BACs, one possessing more than two polymorphic loci, which were then mapped within 0.5 cM in one linkage group. For example, our results found six polymorphic loci produced by SSR primer pairs NAU6520, NAU6530, NAU6593, NAU6658, NAU6675, and NAU6697 from BAC AC202830 all tagged in chromosome D11 (Figure 8). We also found 13 BACs in which more than two polymorphic loci from one BAC were mapped in their homoeologous chromosome pairs. For example, among six polymorphic loci amplified by SSR primers developed from the same BAC (AC188035), two loci amplified by NAU6615 and NAU6667 were tagged in chromosome A10, however, four loci by NAU6215, NAU6476, NAU6515 and NAU6667 tagged in chromosome D10 (Figure 7). Additionally, 11 BACs with more than two polymorphic loci produced by SSR primer pairs from the same BAC were tagged in non-homoeologous chromosomes (for example, two polymorphic loci amplified by NAU6389 and NAU6626 developed from AC190805 were anchored in A4 and D8, respectively (Figure 5, 6)) and 28 BACs in which only one polymorphic locus was tagged (see Additional file 1). Based on these SSR mapping results, however, we could not definitively establish if the mapped polymorphic locus is at exactly the BAC clone position, since these polymorphic loci can be amplified either from the A- or D-subgenome and even other chromosomes in allotetraploid cotton.

#### Identified tagging of 37 BACs based on amplified product analysis

**Figure 10:**
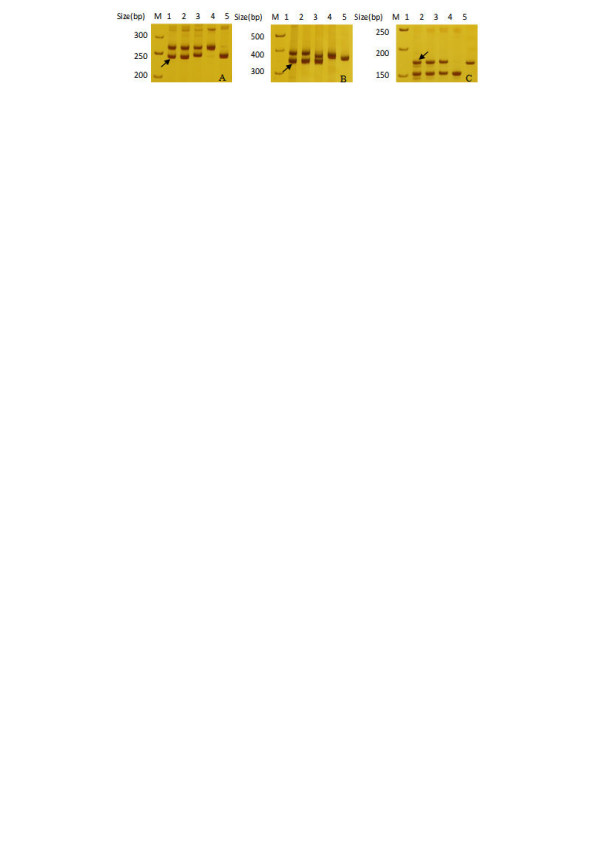
**Identification of three BACs (AC187848, AC190263 and AC188140) belongings by amplified size analysis**. Note: 1. *G. hirsutum *cv. Maxxa; 2. *G. hirsutum *acc. TM-1; 3. *G. barbadense *cv. Hai7124; 4. *G. herbaceum *var. *africanum*; 5. *G. raimondii *Arrow: amplified product with expected size from Maxxa. A: NAU6627 for AC187848 with expected size 247 bp. SSR tagging position was consistent with BAC clone belongings. B: NAU6202 for AC190263 with expected size 349 bp. BAC clone belongings is in homoeologous chromosome of SSR tagging position. C: NAU6465 for AC188140 with expected size 171 bp. BAC clone belongings is confirmed by comparing amplified product size in tetraploid with that in their diploid progenitors *G. herbaceum *and *G. raimondii*.

We are left to question if we can exactly anchor these BACs into their corresponding chromosomes by their-derived SSR markers. It is well known that allotetraploid cotton contains two distinct genomes, which resemble the extant A-genome of *G. herbaceum *(n = 13) and D-genome of G. *raimondii *Ulbrich (n = 13). The A- and D-genome species diverged from a common ancestor approximately 6–11 million years ago. Therefore, most SSR primers should easily amplify two loci, one from At- and the second from Dt-subgenome chromosomes. So, if one polymorphic locus was detected and mapped between Hai7124 and TM-1, and the amplified product was the same as that in Maxxa (from which BAC clones were isolated and SSRs developed), we concluded that this SSR derived-BAC should be anchored at its SSR tagging position. For example, SSR primer NAU6627, derived from AC187848, generated two loci from *G. hirsutum *acc. TM-1, and *G. barbadense *cv. Hai7124 (Figure 10A). One locus produced two polymorphic alleles, NAU6627_-250 _in Hai7124 and NAU6627_-247 _in TM-1, and mapped on chromosome D11 (Figure 8, Additional file 1). NAU6627 was designed based on sequence information from AC187848 in Maxxa and its expected product size was 247 bp in Maxxa, therefore we concluded that the BAC was anchored into chromosome D11 in the D-subgenome. Following further analysis of amplified products from primer NAU6627 in diploid species *G. herbaceum *var. *africanum *and *G. raimondii*, (the two closest extant relatives of the original tetraploid progenitors), we could still anchor this polymorphic locus into the D-subgenome, given *G. raimondii *produced almost the same fragment at the expected 247 bp product size. Furthermore, two loci were amplified from each TM-1 and Hai7124, with one monomorphic locus producing the expected product size from the BAC clone in Maxxa and the other a polymorphic locus tagged in the genetic map. Therefore, we associated this BAC clone with the subgenome chromosome by tagging comparisons of the polymorphic locus and the amplified products from *G. herbaceum *var. *africanum *and *G. raimondii*. The SSR primer NAU6202 derived from AC190263 generated two loci in both TM-1 and Hai7124 (Figure 10B), validating the former results. One SSR locus was monomorphic, the alleles approximately 350 bp in size, and the other locus produced two polymorphic alleles, NAU6202_-380 _in Hai7124 and NAU6202_-400 _in TM-1, which subsequently mapped on chromosome A7 (Figure 6, Additional file 1). Since NAU6202 was designed based on sequence information from AC190263 in Maxxa and its expected product size was 349 bp, close to the monomorphic allele size in *G. raimondii*, we concluded that the BAC was anchored into the D-subgenome. Finally, two loci were amplified by SSR primer pairs, which exhibited the same molecular size between TM-1 (Maxxa) and Hai7124 for each locus. In addition, the two loci were individually amplified in their diploid progenitors *G. herbaceum *and *G. raimondii *with almost the same fragment size as in the corresponding tetraploid subgenome. The sub-genome composition of the amplified product was confirmed by comparing its product with diploid *G. herbaceum *and *G. raimondii *(Figure 10C). For example, NAU6465 SSR primer pairs amplified two monomorphic loci with product sizes of 170 bp and 150 bp, respectively in TM-1 and Hai7124. Two fragments of 150 bp and 170 bp respectively from diploid *G. herbaceum *and *G. raimondii *were also generated. NAU6465 SSR primer pairs were developed based on sequence information from AC188140 in Maxxa with an expected size of 171 bp (Additional file 1). *G. raimondii *produced a 170 bp fragment very near the expected 171 bp. Therefore, we determined that the AC188140 clone was anchored in the D-subgenome.

According to the criteria described above, 37 clones were truly anchored to the A- and D-subgenomes. Twelve BACs belonged to the A-subgenome and 25 to the D-subgenome (Table 4). The remaining 33 BACs could not be anchored to their subgenome because two distinct amplified loci corresponding to A- or D- subgenome were not resolved in tetraploid cotton. Therefore, the need for further experimentation, such as BAC-FISH analysis was recommended. Interestingly, among 25 BACs belonging to the D-subgenome, 19 BAC taggings coincided with SSR mapping. However, among 12 BACs within the A-subgenome, four (AC187470; AC187836; AC194383 and AC202821) were not consistent with SSR mapping. The four BACs were all anchored on the A-subgenome, but more than two polymorphic SSR loci from the same BAC were mapped on D-subgenome chromosomes (Table 4, Additional file 1). These results indicated that different evolutionary pressures acted on the A-subgenomes and D-subgenomes among different tetraploid cotton species in their corresponding homoeologous loci, and that the D-subgenomes exhibited more rapid evolutionary rates with increased nucleotide and allelic diversity than the A-subgenomes.

**Table 4:** The subgenome belongings of BAC clones

BACs	Chro. Tagged*	Subgenome**	BACs	Chro. Tagged*	Subgenome**
AC187225	A2/D2	A	AC187200	D13	D
AC187470	D11	A	AC187214	D11	D
AC187478	A10/D10	A	AC187471	D6	D
AC187836	D5	A	AC187578	A7/D7	D
AC188017	A12/D12	A	AC187810	D11	D
AC188842	A2/D2	A	AC187794	D11	D
AC193383	A11	A	AC187848	D11	D
AC194364	A13/D13	A	AC188026	D9	D
AC194383	D10	A	AC188035	D10	D
AC202821	D11	A	AC188140	D10	D
AC202822	A9	A	AC188200	D10	D
AC193751	unknown	A	AC188401	D8	D
			AC188760	D2	D
			AC189748	D3	D
			AC190263	A7/D7	D
			AC190279	D9	D
			AC193505	A5/D4	D
			AC193514	D1	D
			AC193940	D9	D
			AC193994	D6	D
			AC197182	A5/D5	D
			AC202830	D11	D
			AC202831	D5	D
			AC187545	unknown	D
			AC187548	unknown	D

*The chromosome tagging position of polymorphic SSR loci that SSR primer pairs were developed from corresponding BAC clone.

** The subgenome belongings of corresponding BAC clone.

#### Comparative sequence analysis between the A- and D-subgenome chromosomes

Thirty-seven BACs with verified origins were identified in this study, 12 BACs belonging to the A-subgenome with a total length of 1,200,814 bp length including 69 gaps (average 5.75 gaps/BACs); and 25 BACs within the D-subgenome covering 2,374,313 bp length with 37 gaps (average 1.48 gaps/BACs). These results indicated that A-subgenome BACs possessed regions more difficult to sequence than those from the D-subgenome. Furthermore, the genes predicted from the 37 BACs were evaluated for possible intron size contributions that correlated with genome size between the A- and D-subgenome chromosomes. In the 12 BACs belonging to the A-subgenome, 67 genes were predicted with an average of 937 bp exons and 920 bp introns for each gene; however, in the 25 BACs belonging to the D-subgenome, 104 genes were predicted with an average of 1297 bp exons and 1414 bp introns for each gene. Therefore, introns might have no contribution to different subgenome size in *Gossypium*.

## Discussion

### Characteristics of genome structure in allotetraploid cotton

Cotton is the world's most important natural textile fiber and a significant oilseed crop. Cotton fiber is also an outstanding single-cell model to study plant cell elongation, and cell wall and cellulose biosynthesis [26]. Of all 50 cotton species, *Gossypium hirsutum *provides over 95% of the annual cotton crop worldwide. Elucidating the tetraploid cotton genome composition and structure, especially upland cotton, will vastly expand opportunities in cotton research and agronomic improvements worldwide. However, cotton possesses a complex genome so whole genome sequencing of tetraploid cotton represents a substantial challenge [13]. The GeneTrek approach has been proposed as an efficient means to evaluate the general properties of any genome by annotating a small set of randomly selected BACs [19,20]. In maize, sequence analysis of 100 randomly selected BACs led to the prediction of 42,000–56,000 genes with at least 66% repetitive DNA [27]. In addition, sequence analysis of 74 randomly selected BACs showed that the maize nuclear genome contains about 37,000 candidate genes and 5,500 truncated and probable pseudogenes. However, the distribution of genes and repetitive elements is uneven [21]. In the present study, properties of the upland cotton genome, such as total gene number, amount and distribution of repetitive DNA, and gene distribution, were first predicted based on the annotation of 142 randomly sequenced BACs. Compared with a density of one gene every 7.5 kb in the *CesA *region of homoeologous BACs [14], the *AdhA *region of homoeologous BACs exhibits one gene per 20 kb for the A-subgenome and one gene every 13 kb for the D-subgenome [18]. These data led to the prediction of more than 70,000 genes with one gene per 34.5 kb in upland cotton. Because upland cotton is an allotetraploid and has duplicated copies of genes in homoeologous regions of the A- and D-subgenomes, approximately 35,000 genes were predicted in each subgenome. In tetraploid cotton, the distribution of genes is uneven, with gene-rich and gene-free regions. We also found 21% of BACs lacked genes and 72.5% of the gene islands contained only one gene. These results indicated that selecting only gene-rich BACs for cotton genome sequencing is not adequate to cover the entire genome, owing to the fact that more than one fifth of BACs exhibit an absence of genes.

In this study, 1,653 predicted gene models lacked homology to other species in the NCBI protein database. In addition, we verified 208 ESTs by BLASTN queries against the cotton EST database. However, we could not confirm if these transcripts were related to mobile elements, gene candidates, or special products in cotton. Therefore, we have not used the information to predict the structure and composition of the upland cotton genome. However, the functions and properties of these transcripts warrant further study to enhance the understanding of the complex upland cotton genome.

### Structure difference between A- and D-subgenome chromosomes

In plants, the following factors have been summarized as the main mechanisms for genome size expansion: (1) long terminal repeat (LTR) retrotransposable element amplification and insertion such as that in maize [28]; (2) variation in intron size [29]; (3) expansion of tandemly repetitive DNA sequences [30]; (4) segmental duplications [31]; (5) accumulation of pseudogenes [32]; and (6) transfer of organellar DNA to the nucleus [33]. The cultivated cotton species *Gossypium hirsutum *has long been known as an allotetraploid possessing a nuclear A- and D-subgenome. A- and D-genome species diverged from a common ancestor approximately 5–10 Mya and acquired genomes that differ nearly twofold in size [2]. Based on the putative mechanisms of genome size expansion described above, it is uncertain which of the mechanism(s) played an important role in the composition and structure of the tetraploid cotton genomes. To explore this question, several studies have been initiated through comparative sequence analysis of specific genomic regions or by application of more global approaches [14,16,18]. Grover et al. (2004)[14] investigated A- and D-genome size evolution from tetraploid cotton in a 104 kb contiguous sequence surrounding the *CesA1 *gene, and demonstrated no evidence of genome size variation between the A- and D-subgenome genic regions. In a similar study, Grover et al. (2007)[18] obtained the aligned length surrounding the *AdhA *gene with 101.7 kb in the A-subgenome, 49 kb in the D-subgenome, 112.3 kb from the diploid A-genome and 55 kb from the diploid D-genome. The results revealed the aligned length size variation was mainly attributed to differential accumulation of retroelements. Hawkins et al. (2006)[16] compared diploid A- and D-genome size differences by utilizing the whole genome shotgun (WGS) method and concluded that 40%–65% of each genome is composed of transposable elements, with *Copia*-like sequences accumulated in smaller genomes and *Gypsy*-like sequences in larger genomes.

Based on the sequence analysis of 37 subgemone-known BACs, we found no relationship between introns and different subgenome size in *Gossypium*. However, an average of 5.75 gaps/BAC indicated an increased number of gaps, lending difficulty to BAC assembly in the A-subgenome. The D-subgenome had an average of 1.48 gaps/BAC, demonstrating that BACs from the A-subgenome are more difficult for sequence assembly than those from the D-subgenome. This and previous studies revealed the presence of homeolog sequence and structure conservation in gene-rich regions, suggesting large amplification of transposable elements may not be in gene-rich regions, but may reside in low-density gene or gene-free regions. In future studies, the structure and function of DNA sequences in these gap regions can be confirmed by whole BAC sequence assembly analysis; and A-specific and D-specific regions related with transposable elements can be located using combined BAC-FISH technology.

### The D-subgenome has a more rapid evolutionary rate in different tetraploid cotton species

Sequence and marker analyses from several previous studies indicated that varied evolutionary pressures might act on the D-subgenomes from different tetraploid cotton species. In both *G. hirsutum *and *G. barbadense*, the D-subgenome maintained greater nucleotide and allelic diversity than did the A-subgenome, results supported by duplicated paralogous *Adh *loci comparisons [34,35]. In addition, *G. raimondii*-derived EST-SSR markers had high polymorphic frequencies between *G. hirsutum *and *G. barbadense *[25]. In this paper, we investigated whether BACs were characterized by an A- or D-subgenome. SSR marker BACs were largely tagged in the D-subgenome determined by integration of polymorphic marker loci with our tetraploid cotton backbone linkage groups. Our results further confirmed previous studies where sequence and structure conservation of homeologs between the A- and D- subgenomes was high. These data are consistent with the evolutionary history of tetraploid cotton progenitors, where diploid A- and D-genome species were derived from the same ancestor approximately 5–10 Mya. Alternatively, relaxed selection acted on the D-subgenomes from different tetraploid cotton species, evidenced by greater DNA sequence diversity among D-subgenomes than A-subgenomes in different tetraploid cotton species.

## Conclusion

The study provided us the first glimpse at cotton genome complexity, and the results indicated that the gene distribution in cotton genome is uneven with gene-rich and gene-free regions, and rich in repetitive elements. This study will serve as a foundation for tetraploid cotton whole genome sequencing in the future.

## Methods

### Cotton BACs

One hundred forty-five cotton BAC sequences were downloaded from the National Center for Biotechnology Information (NCBI) [22] on June 2, 2007. As part of the maize sequencing project by the Genome Sequencing Center, Washington University School of Medicine, the BACs were initially submitted as *Zea mays*. However, further analysis, determined the clones were from *Gossypium hirsutum *cv. Maxxa. The sequence data used in this paper were the product of collaborative efforts by The Maize Sequencing Consortium, including the University of Arizona, Cold Spring Harbor Laboratory, Iowa State University, and the Genome Sequencing Center at Washington University School of Medicine in St. Louis. We selected 142 from 145 BACs with sizes > 20 kb for gene annotation. A 32,101 bp length gap region from AC189045 was later excluded because the predicted genes were phage related and it was decided the sequence data were contaminated. Finally, 142 BACs spanning nearly 14.2 Mb (0.5%) of the cotton genome were used for the analysis.

### Genetic mapping of BAC clones based on the simple sequence repeats (SSRs)

Each BAC was searched for SSRs with the online software SSRIT [36]. SSRIT, written in Perl script, is a microsatellite search tool available at the USDA-ARS Center for Bioinformatics and at Comparative Genomics at Cornell University. Dinucleotide, trinucleotide, tetranucleotide, pentanucleotide and hexanucleotide SSRs were detected with SSRIT. The search standards for different repeat motifs were as described in Wang et al. (2006) [37]. Primer pairs flanking the SSRs were designed using the program Primer3.0 [38] and tested against our mapping parents, *G. barbadense *cv. Hai7124 and *G. hirsutum *acc. TM-1, standard lines for genetic and genomic research. Furthermore, the polymorphic SSRs were integrated into our backbone genetic map of allotetraploid cultivated cotton [25] using Joinmap 3.0 software with a minimum log-of-odds (LOD) score of 6.0. The structure of known BACs was further identified using mapping results and molecular size comparisons among *G. hirsutum *cv. Maxxa, *G. hirsutum *acc.TM-1, and *G. barbadense *cv. Hai7124, with diploid *G. herbaceum *var. *africanum *and *G. raimondii *as controls.

### Annotation of LTR retrotranspons and other mobile elements

Repetitive element prediction was accomplished through Repeatmasker [39], CENSOR [40], and BLAST identity to characterize elements in REPBASE (version 8.5) [41]. Compared with the results of repetitive element prediction, LTR retrotranspons were further identified by LTR_finder software [42], and manually verified by structural features such as LTR and TSD pairs, a primer binding site and a polypurine tract.

### Sequence analysis and gene annotation

BAC sequences were subject to three *ab initio *gene prediction programs, FGENESH (Softberry) [43], GENSCAN+[44] and GENEMARK.HMM [45]. Gene models provided query sequences to search the National Center for Biotechnology Information (NCBI) non-redundant protein database and the *Arabidopsis thaliana *protein database [46]. All BLASTP hits were manually evaluated to determine if a gene model was likely to be a real gene or not based on *e*-value, query alignment and hit annotation. The integral parts of known repetitive elements were removed from the above gene models for further analysis. In addition, the sequences with gene models but no annotations were subjected to BLASTN queries against the cotton EST database released in the NCBI website [47]. Gene Ontology (GO) of tandem duplication genes was obtained from UniProt Gene Ontology [48]. The GO values for the best homologous hits were used to determine the ontology of molecular function, cellular components and biological processes for these sequences.

## Abbreviations

BAC: Bacterial Artificial Chromosome; FISH: Fluorescence In Situ Hybridization; QTL: Quantitative Trait Loci; LTR: Long Tandem Repeat; TSD: Target Site Duplications; SSR: Simple Sequence Repeat; WGS: Whole Genome Shotgun.

## Authors' contributions

Experiments were designed by WZG with suggestions from TZZ. Experiments were performed by WZG and CPC. Bioinformatics analyses were performed by CBxW. LZ and LW helped with data analyses. WZG drafted the manuscript and TZZ revised the manuscript. All authors read and approved the final manuscript.

## Supplementary Material

Additional file 1 Summary of SSR primers developed from BACs generated from *Gossypium hirsutum *cv. Maxxa. The data provided all the markers' information developed from 142 BACs generated from *Gossypium hirsutum *cv. Maxxa. Click here for file

Additional file 2 Gene features predicted in 142 cotton BACs. The data provided all gene features predicted in 142 cotton BACs. Click here for file

Additional file 3 142 BACs clones information. The data provided 142 BACs clones information in detail. Click here for file

Additional file 4 Tandem duplication genes in 30 gene islands. The data provided the ontology analysis of tandem duplication genes in 30 gene islands. F: molecular function; C: cellular component; P: biological process. Click here for file
